# Public health impact of strain specific immunity to *Borrelia burgdorferi*

**DOI:** 10.1186/s12879-015-1190-7

**Published:** 2015-10-26

**Authors:** Camilo E. Khatchikian, Robert B. Nadelman, John Nowakowski, Ira Schwartz, Michael Z. Levy, Dustin Brisson, Gary P. Wormser

**Affiliations:** Evolution and Ecology of Disease Systems Laboratory, Department of Biology, University of Pennsylvania, Philadelphia, PA USA; Division of Infectious Diseases, Department of Medicine, New York Medical College, Valhalla, NY USA; Department of Microbiology and Immunology, New York Medical College, Valhalla, NY USA; Department of Biostatistics and Epidemiology, University of Pennsylvania Perelman School of Medicine, Philadelphia, PA USA

**Keywords:** Immunity, Epidemiological impact, Lyme disease, *Borrelia burgdorferi*

## Abstract

**Background:**

Lyme disease, caused by *Borrelia burgdorferi*, is the most common tick-borne infection in the United States. Although humans can be infected by at least 16 different strains of *B. burgdorferi,* the overwhelming majority of infections are due to only four strains. It was recently demonstrated that patients who are treated for early Lyme disease develop immunity to the specific strain of *B. burgdorferi* that caused their infection. The aim of this study is to estimate the reduction in cases of Lyme disease in the United States that may occur as a result of type specific immunity.

**Methods:**

The analysis was performed based on three analytical models that assessed the effects of type specific immunity. Observational data on the frequency with which different *B. burgdorferi* strains cause human infection in culture-confirmed patients with an initial episode of erythema migrans diagnosed between 1991 and 2005 in the Northeastern United States were used in the analyses.

**Results:**

Assuming a reinfection rate of 3 % and a total incidence of Lyme disease per year of 300,000, the estimated number of averted cases of Lyme disease per year ranges from 319 to 2378 depending on the duration of type specific immunity and the model used.

**Conclusion:**

Given the assumptions of the analyses, this analysis suggests that type specific immunity is likely to have public health significance in the United States.

**Electronic supplementary material:**

The online version of this article (doi:10.1186/s12879-015-1190-7) contains supplementary material, which is available to authorized users.

## Background

Lyme disease, caused by *Borrelia burgdorferi*, is the most common tick-borne infection in the United States. Additionally, recent evidence suggests that the 30,000 reported cases actually represent only 10 % of the true incidence in the United States [1]. The majority of reported Lyme disease cases originate in the Northeastern (82 %) and Midwest (9 %) United States [2], although the geographic range is currently expanding [3–5]. Despite the fact that more than 16 *B. burgdorferi* lineages exist in these areas, the majority of disseminated human infections are caused by just four. Similarly, the subsets of *B. burgdorferi* lineages that cause infection in wild animals vary among animal species [6–8].

*B. burgdorferi* lineages have been categorized by their allele at the highly variable outer surface protein C (*ospC*) locus, and are often referred to by their *ospC* genotype. While there is ample evidence that the surface exposed OspC lipoprotein is necessary for infection, and that there is a strong and protective immune response against OspC [9–11], there is currently limited direct evidence that the variability at *ospC* directly affects differential infectivity of humans or other animal species. Nevertheless, the variability at the *ospC* locus, along with the high linkage among all genetic loci [12–15], makes *ospC* a good marker to delineate different *B. burgdorferi* lineages.

Based on a unique data set of culture-confirmed initial and repeat episodes of early Lyme in antibiotic-treated patients with erythema migrans, two conclusions were made. One was that recurrence of early Lyme disease in such patients is due to reinfections rather than relapses of the original infection [16]. Secondly, based on multiple modeling techniques and using observed data on the frequency with which different strains of *B. burgdorferi* were cultured from initial versus recurrent episodes of early Lyme disease, it was demonstrated that type specific immunity lasting at least five years is likely to develop following infection [17].

In this analysis, we attempt to estimate the reduction in cases of Lyme disease that may occur as a result of the recently demonstrated type specific immunity. The analyses suggest that the presence of type specific immunity is likely to have public health significance.

## Methods

Several assumptions were made to perform the analyses. The probability of exposure to various strains of *B. burgdorferi* due to a bite from an infected tick was estimated based on the frequency with which different strains of *B. burgdorferi* were recovered from skin biopsy samples of patients with early Lyme disease associated with erythema migrans in the Lower Hudson Valley region of New York State (Table 1) [17, 18]. These published data were derived from the distribution of strains cultured from the skin of a sample of 200 patients (from a total of 298) with no prior history of Lyme disease that were diagnosed between 1991 and 2005, as described previously [17, 18]. *B. burgdorferi* lineages were previously classified according to the allele at the *ospC* gene as previously described e.g., [15, 18, 19]. The present analysis was performed in 2014 and is exempted from Institutional Review Board (IRB) approval as it involved the analyses of existing published data (category 4), as defined by the Office for Human Research Protections (www.hhs.gov/ohrp/).

**Table 1:** Frequency of the different OspC types that were cultured from the skin of 200 patients with erythema migrans [17, 18].

OspC type	Number (%)
Total	200 (100 %)
**K**	73 (36.5 %)
**A**	38 (19 %)
**B**	28 (14 %)
**I**	14 (7 %)
N	13 (6.5 %)
E	13 (6.5 %)
U	9 (4.5 %)
H	5 (2.5 %)
C	2 (1 %)
D	2 (1 %)
F	1 (0.5 %)
G	1 (0.5 %)
M	1 (0.5 %)
J	0 (0 %)
L	0 (0 %)
T	0 (0 %)

Bold font indicates invasive OspC types, comprising 76.5 % of the total cases

Additional assumptions that were made included that type specific immunity follows infection with all strains of *B. burgdorferi* and is absolute, lasts ≥ 5 years, and is restricted to a single OspC genotype. Based on our prior observations we assumed that 3 % of Lyme disease cases are due to reinfections, but also assessed the impact of type specific immunity if 1 % or 5 % of infections occur as a result of a reinfection.

### Number of *B. burgdorferi* infections averted due to strain specific immunity

The potential impact that strain-specific immunity could have on human Lyme disease incidence was assessed using three different mathematical modeling structures. Analytical models are necessary to estimate what *would* have occurred in the absence of a factor such as strain-specific immunity, a process known as precluding. The models employed account for the total number of human cases as well as the number of averted cases. In this study, the term “averted cases” refers to cases that would have occurred in the absence of strain-specific immunity, holding all other model variables constant.

The three analytical models have different underlying assumptions, different sets of limitations, and different degrees of realism, and are thus useful to assess varying aspects of the effects of strain-specific immunity (Table 2).

**Table 2:** Summary of the characteristics of the three models used to estimate the number of averted cases of Lyme disease due to type specific immunity

Model	Key assumptions^a^	Benefits	Limitations
Deterministic probability	Lyme disease patients’ probability of exposure to infectious bite similar to general population.	Extremely simple and flexible. Allows a separate analysis focusing on infections caused by invasive strains of *B. burgdorferi.*	May over estimate the impact of immunity on averted cases.
	Immunity is permanent.		
		Provides the upper limit of averted cases.	
Equilibrium dynamic	Lyme disease patients’ probability of exposure to infectious bite similar to general population.	Simple.	May under estimate the impact of immunity on averted cases.
		Provides the lower limit of averted cases.	
	Immunity lasts 5 to 30 years.		
	Lyme disease patients are at risk for tick bites for 30 years.		
Individual-based stochastic	Lyme disease patients’ probability of exposure to infectious bite higher than in general population.	Most complex, allows manipulation of many parameters.	Simulations are time-demanding.
		May provide the most realistic estimate of the number of averted cases.	
	Immunity lasts 5 to 30 years.		
	Patients are at risk for tick bites for 30 years.		

^a^all models share the key assumptions that immunity provides 100 % protection to a particular OspC type of *B. burgdorferi*, that there is no cross-immunity across different OspC types, and that in the absence of immunity the likelihood of developing infection with a particular OspC type follows the strain frequencies presented in Table 1

### Deterministic probability model

With the deterministic probability model the proportion of human cases that are averted due to strain-specific immunity is equivalent to the proportion of patients that are exposed to a *B. burgdorferi* strain with which the patient has been previously infected. This proportion can be calculated as

$$ A=\varSigma {f_i}^2 $$

where *f*_*i*_ is the frequency of the *i*^th^ strain (Table 1) and *A* is the proportion of cases averted due to strain-specific immunity; eg., the proportion of patients that are exposed to strain K on two separate occasions occurs at a frequency of *f*_*K*_^*2*^ = 0.365^2^ = 0.1332 (Table 1). Based on the ~30,000 Lyme disease reported cases in the United States and an estimated 3 % reinfection rate the number of averted cases can be calculated as

$$ N=\left(0.03\ *A\right)/\left(1 - A\right)\ *\ 30,000 $$

where *N* is the total number of cases averted in relationship to the total number of cases in the United States.

### Equilibrium dynamic model

In the equilibrium dynamic model, humans can change from susceptible (S) to infected (I) to immune (R) and back to susceptible (S). The effect of strain-specific immunity on human Lyme disease incidence is the sum of number of averted cases from exposure to each individual *B. burgdorferi* strain.

Patients transition from susceptible to strain *i* (*S*_*i*_) to infected by strain *i* (*I*_*i*_) at rate β_*i*_, equivalent to the incidence rate of strain *i*.

$$ {I}_i={\upbeta}_{\mathrm{i}}*\ {S}_i $$

The incidence rate for each strain *i* is scaled to the total incidence rate for all *B. burgdorferi* strains using the frequency at which each strain is found in human infections such that

$$ \beta = \varSigma \kern0.5em {\beta}_i $$

Patients transition from the infected state (*I*_*i*_) to the immune state (*R*_*i*_) at a rate of 1, such that the transition occurs with each iteration of the model (1 year) with 100 % probability. Patients are assumed to transition from the immune state (*R*_*i*_) back to the susceptible state (*S*_*i*_) in either 5 years (γ = 0.2) or 30 years (γ = 0.0333). These 2 limits were selected based on prior estimates of the minimum duration of strain specific immunity [17] or a maximum approximating life-long immunity. Therefore, the number of averted cases of Lyme disease can be estimated as the number of individuals in the infected or immune states (*I*_*i*_ + *R*_*i*_) that are exposed to strain *i.*

$$ A={\beta}_i*\ \left({I}_i+{R}_i\right) $$

where *A* is the number of averted cases as a function of the incidence rate of Lyme disease (β_*i*_).

### Individual-based stochastic simulation model

In the stochastic simulation model, we simulated 100,000 individuals over a 30 year period in which they could be exposed to an infectious tick bite. Each individual has a set per year probability of exposure to an infectious tick bite which is drawn from an exponential frequency distribution such that most individuals have a very low per year probability of exposure. The rate parameter of the exponential distribution was chosen such that the reinfection rate would equal 3 % of total infections at an incidence rate of 100 cases of Lyme disease per 100,000 people.

In each year that an individual is available to be exposed to an infectious tick bite, the simulation determines if the individual is exposed to an infectious tick bite given the per year probability of exposure of the simulated individual and a random number generator. If the individual is exposed to an infectious tick bite, the probability of exposure to a particular borrelial strain was assumed to equal the frequency at which that strain has been recovered from patients (Table 1). If the simulated individual has been infected by the chosen strain within the time frame of strain-specific immunity, the individual is considered immune and an averted case is recorded. If the individual has not been previously infected or if the duration of strain-specific immunity has ended, an infection with the chosen strain is recorded. The simulation records the number of averted and successful infections among 100,000 simulated individuals. For every combination of parameters, 200 simulations were run each with 100,000 individuals. Parameters included varying duration of strain-specific immunity (5 or 30 years) and varying incidence rates of Lyme disease (0–500 per 100,000 people).

## Results

If the reinfection rate is 3 % and the total number of Lyme cases per year is 300,000, the estimated number of averted cases of Lyme disease per year ranges from 319 to 2,378 depending on the model employed and the particular assumptions used for that model (Table 3). With different assumptions on the incidence of Lyme disease and on the frequency of reinfections, however, the number of averted cases per year might be as few as 11 or as many as 4,100 (Table 3 and Additional file 1: Figure S1).

**Table 3:** Estimation of the yearly number of averted Lyme diseases cases in the United States based on the number of Lyme disease cases reported (approximately 30,000) or estimated (around 300,000) using three different analyses. The number of averted cases was calculated using three different estimates of reinfection rates: 1 %, 3 %, and 5 %

Lyme disease incidence	Immunity length	Estimated number of averted cases		
		Deterministic probability model^a^		
		1 % reinfection	3 % reinfection	5 % reinfection
30,000 cases	life span	77	232	387
300,000 cases	life span	775	2,324	3,873
		Equilibrium dynamic model		
		1 % reinfection	3 % reinfection	5 % reinfection
30,000 cases	5 years	11	32	53
30,000 cases	30 years	78	233	390
300,000 cases	5 years	106	319	532
300,000 cases	30 years	776	2,333	3,898
		Individual-based stochastic model		
		1 % reinfection	3 % reinfection	5 % reinfection
30,000 cases	5 years	23	55	80
30,000 cases	30 years	90	238	410
300,000 cases	5 years	227	549	799
300,000 cases	30 years	899	2,378	4,100

^a^Calculations based on the values in Table 1

Available published data suggest that 83.3 % of patients with blood culture-confirmed disseminated *B. burgdorferi* infection from the Lower Hudson Valley region of New York State are infected with one of just four OspC genotypes: A (accounted for 23.5 % of positive blood cultures), B (for 14.4 %), I (for 12.1 %) or K (for 33.3 %) [18]. Assuming that a patient was initially infected by one of these four strains and that patients are exposed to *B. burgdorferi* strains at rates consistent with published data on initial human infections (Table 1, Additional file 2: Table S1), the results of the deterministic model suggest that the probability of a positive blood culture in patients with a recurrence of erythema migrans would be reduced by approximately 25 % due to strain-specific immunity.

## Discussion

The number of cases of Lyme disease in the United States continues to rise over time and expand geographically [3, 5]. Prevention methods rely primarily on measures to avoid tick bites, which have had variable rates of adherence and success [20]. Unfortunately, no vaccine to prevent *B. burgdorferi* infections is currently available for humans.

The results of this study suggest, given the assumptions of an annual 3 % reinfection rate and 300,000 total cases per year, that were it not for the presence of type specific immunity there might be as many as 2,300 additional cases of Lyme disease per year in the United States. Although only recently recognized [17], the presence of type specific immunity to strains of *B. burgdorferi* is therefore likely to have public health significance. Furthermore, the frequency of Lyme disease cases with hematogenous dissemination of the spirochete would also be appreciably higher in the absence of type specific immunity. Our findings suggest a 25 % risk reduction in having a positive blood culture during a recurrence if the initial infection was caused by one the four genotypes most often associated with detectable spirochetemia. In a previously published study the frequency of a positive blood culture for *B. burgdorferi* in 41 patients with a prior history of Lyme disease was 31.7 % versus 46.5 % in 172 patients during a first episode of Lyme disease [18]. Thus, the observed 31.5 % reduction in the rate of positive blood cultures in patients with prior Lyme disease in that study is consistent with the existence and clinical relevance of type specific immunity.

The public health effects of type specific immunity have been previously observed with several pathogens. For example, patients infected with one variety of the influenza virus are protected from reinfection with the same strain, but remain susceptible to other strains. The public health effect of type specific immunity in human influenza has temporal limitations due to the rapid evolution of novel strains [21], which is not apparent in *B. burgdorferi* populations.

Limitations of our study center around the accuracy of several key assumptions: that Table 1 reflects the overall frequency of infection by different strains of *B. burgdorferi* in the United States, that type specific immunity is restricted to just a single genotype, and that type specific immunity is 100 % protective for that genotype. The reported frequencies at which humans are exposed to each *B. burgdorferi* strain (Table 1) may be inaccurate due to geographic variation in the frequencies of strains in ticks and due to differences in cultivability among strains. The frequencies of *B. burgdorferi* strains in ticks differs among northeastern, Midwestern, and California Lyme disease foci e.g.,[6, 15, 22, 23] implying that human exposure rates differ [24]. These differences would alter the calculations of the public health effect of type specific immunity for the 18 % of infections that occur outside of the northeastern foci [2], although it is difficult to predict if the calculated number of averted cases would increase or decrease from those reported here.

Inaccuracies in the reported frequencies at which humans are exposed to each *B. burgdorferi* strain (Table 1) may also be caused by culture biases. However, several lines of evidence suggest that it is unlikely that culture bias has resulted in the uneven detection of strains in human blood. First, there are differences in the frequencies of strains in cultures of skin versus blood of humans. Second, non-culture methodologies performed with various animal species produce similar types of frequency biases, suggesting that the biases are not the result of differences in cultivability. In addition to culture biases, we did not consider in the analyses that ticks may transmit more than one strain of *B. burgdorferi* simultaneously [6, 19], and thus even if the person bitten was immune to reinfection by one of the transmitted strains, that individual might still develop Lyme disease from the other transmitted strain(s).

The accuracy of the assessment of the public health impact of type specific immunity is also impacted by the assumption that type specific immunity is restricted to just a single genotype and that it is 100 % protective for that genotype. The analyses assumed that each strain elicited a protective response against infections with the same strain but afforded no cross protection for other strains. It is possible that partial or full protective immunity also develops against additional strains [25], which would elevate the estimate of the public health effect of this type of immunity. The analyses also assumed 100 % protection from reinfection. Relaxing this assumption would decrease the estimate of the public health impact of type specific immunity. Experimental infections or data sets with larger numbers of patients with recurrent infections are necessary to determine the extent of cross immunity among strains and the degree of protection.

Although none of these assumptions is likely to be completely accurate, making a precise estimate of the impact of type specific immunity impossible to determine, it is reasonable to conclude that the presence of some degree of type specific immunity should serve to reduce the number of recurrent infections. In addition, the impact of immunity on Lyme disease prevention is likely to be higher than we have estimated, since the immune response associated with later manifestations of Lyme disease, such as Lyme arthritis, was not considered in this analysis and is likely to be broader and longer lasting than would occur following erythema migrans [26].

The molecular mechanisms leading to type specific immunity in *B. burgdorferi* are not known. Immune responses targeting OspC have been the focus of considerable research, but evidence to support the hypothesis that an immune response to OspC is responsible for type specific immunity is indirect. For example, transient immunity to reinfection with the same strain of *B. burgdorferi* has been demonstrated in laboratory animals and mice immunized with one OspC protein are selectively protected against infection with only *B. burgdorferi* expressing the same OspC protein, not against strains expressing a different OspC protein [27, 28]. Strain-specific immunity is unlikely to be restricted to OspC and may develop against multiple *B. burgdorferi* strain-specific proteins that are in genetic linkage with OspC e.g., [13, 19, 29]. Further empirical studies that characterize multiple antigens are necessary to determine what antigens are responsible for strain-specific immunity.

## Conclusion

Recent data have demonstrated the development of type specific immunity against the infecting strain of *B. burgdorferi* in patients treated for early Lyme disease. The current analysis suggests that this observation is likely to have public health significance.

## Additional files

Additional file 1: Figure S1. The proportion of cases occurring in patients with a prior infection and the proportion of averted cases increases exponentially with higher incidence rates in both the equilibrium dynamic (left column, panels A, C) and individual stochastic models (right column, panels B, D). The proportion of reinfections that are averted due to strain-specific immunity (bottom row, panels E, F) is constant across incidence rates in both models. The dashed lines describe the data output when strain-specific immunity is assumed to last 5 years; the black lines describe the data output when strain-specific immunity is assumed to last 30 years. (DOC 311 kb) Additional file 2: Table S1. Frequency of the different OspC types that were cultured from the skin of 200 patients with erythema migrans (17, 18) and expected percentage increase in total reinfections due to particular OspC types, if there were no strain specific immunity based on the deterministic probability model. Bold font indicates invasive OspC types, comprising 76.5 % of the total cases. (DOC 35 kb)
