# Supplementary material for: Public health impact of strain specific immunity to Borrelia burgdorferi
Source: BMC Infect Dis. 2015 Oct 26;15:472. doi: 10.1186/s12879-015-1190-7 (PMC4621928; doi:10.1186/s12879-015-1190-7)

Supplementary Fig. 1: The proportion of cases occurring in patients with a prior infection and the proportion of averted cases increases exponentially with higher incidence rates in both the equilibrium dynamic (left column, panels A, C) and individual stochastic models (right column, panels B, D). The proportion of reinfections that are averted due to strain-specific immunity (bottom row, panels E, F) is constant across incidence rates in both models. The dashed lines describe the data output when strain-specific immunity is assumed to last 5 years; the black lines describe the data output when strain-specific immunity is assumed to last 30 years.


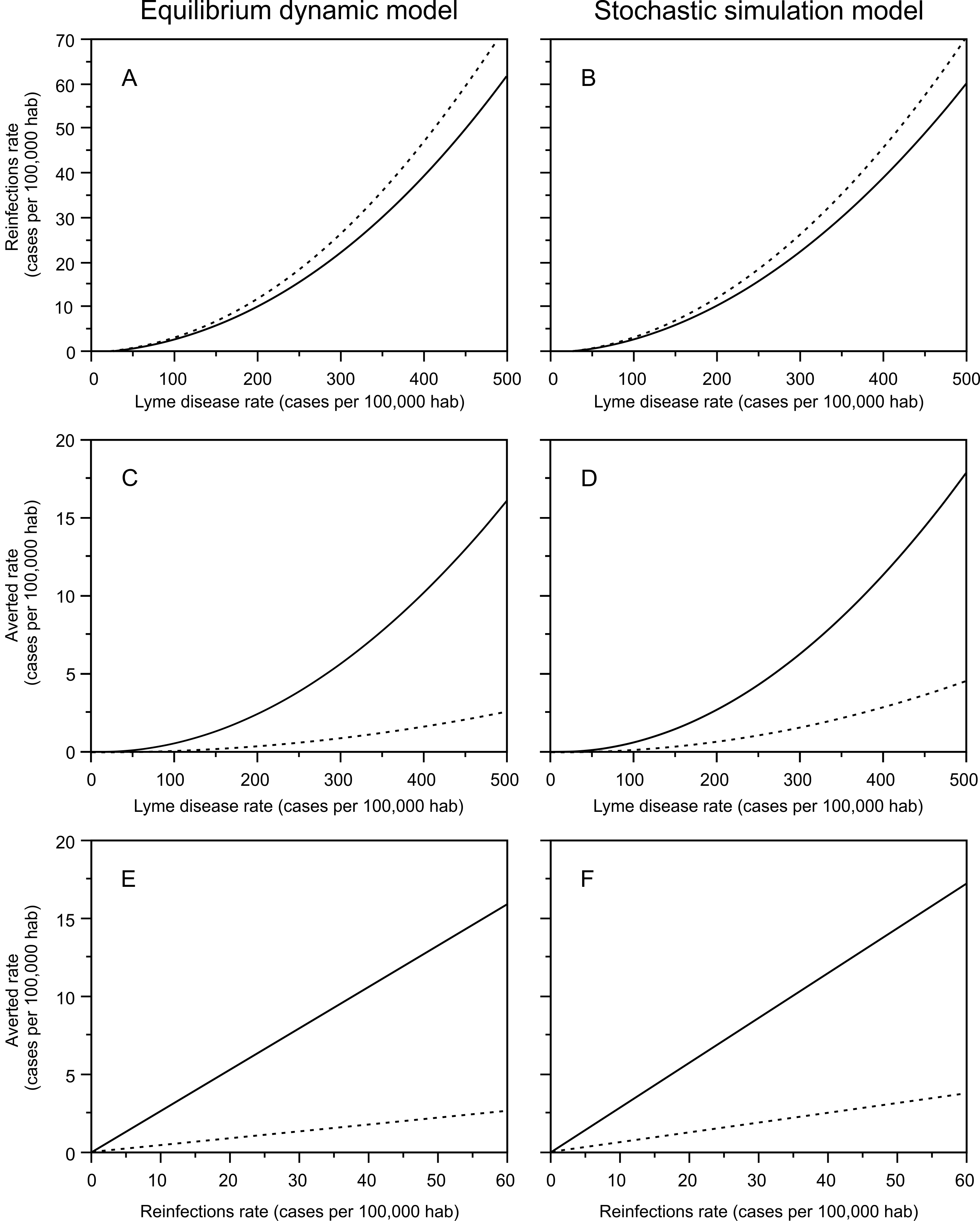

Supplement: Additional file 1: Figure S1. — The proportion of cases occurring in patients with a prior infection and the proportion of averted cases increases exponentially with higher incidence rates in both the equilibrium dynamic (left column, panels A, C) and individual stochastic models (right column, panels B, D). The proportion of reinfections that are averted due to strain-specific immunity (bottom row, panels E, F) is constant across incidence rates in both models. The dashed lines describe the data output when strain-specific immunity is assumed to last 5 years; the black lines describe the data output when strain-specific immunity is assumed to last 30 years. (DOC 311 kb) [file 12879_2015_1190_MOESM1_ESM.doc]
