# Supplementary material for: Public health impact of strain specific immunity to Borrelia burgdorferi
Source: BMC Infect Dis. 2015 Oct 26;15:472. doi: 10.1186/s12879-015-1190-7 (PMC4621928; doi:10.1186/s12879-015-1190-7)
Supplement: Additional file 2: Table S1. — Frequency of the different OspC types that were cultured from the skin of 200 patients with erythema migrans (17, 18) and expected percentage increase in total reinfections due to particular OspC types, if there were no strain specific immunity based on the deterministic probability model. Bold font indicates invasive OspC types, comprising 76.5 % of the total cases. (DOC 35 kb) [file 12879_2015_1190_MOESM2_ESM.doc]

Supplementary Table 1. Frequency of the different OspC types that were cultured from the skin of 200 patients with erythema migrans (17, 18) and expected percentage increase in total reinfections due to particular OspC types, if there were no strain specific immunity based on the deterministic probability model. Bold font indicates invasive OspC types, comprising 76.5% of the total cases.

| OspC type | Number (%) | Expected increase (%) |
| --- | --- | --- |
| Total | 200 (100%) | 22.75 |
| **K** | 73 (36.5%) | 15.37 |
| **A** | 38 (19%) | 3.75 |
| **B** | 28 (14%) | 2.00 |
| **I** | 14 (7%) | 0.49 |
| N | 13 (6.5%) | 0.42 |
| E | 13 (6.5%) | 0.42 |
| U | 9 (4.5%) | 0.20 |
| H | 5 (2.5%) | 0.06 |
| C | 2 (1%) | 0.01 |
| D | 2 (1%) | 0.01 |
| F | 1 (0.5%) | 0.00 |
| G | 1 (0.5%) | 0.00 |
| M | 1 (0.5%) | 0.00 |
| J | 0 (0%) | 0.00 |
| L | 0 (0%) | 0.00 |
| T | 0 (0%) | 0.00 |
